# Neonatal *Streptococcus pneumoniae* Infection May Aggravate Adulthood Allergic Airways Disease in Association with IL-17A

**DOI:** 10.1371/journal.pone.0123010

**Published:** 2015-03-27

**Authors:** Baohui Yang, Ru Liu, Ting Yang, Xiaoli Jiang, Liqun Zhang, Lijia Wang, Qinghong Wang, Zhengxiu Luo, Enmei Liu, Zhou Fu

**Affiliations:** 1 Ministry of Education, Key Laboratory of Child Development and Disorders, Key Laboratory of Pediatrics in Chongqing, Chongqing International Science and Technology Cooperation Center for Child Development and Disorders, Chongqing, China; 2 Department of Respiratory Medicine, Children's Hospital, Chongqing Medical University, Chongqing, China; 3 The Central Laboratory of Children's Hospital, Chongqing Medical University, Chongqing, China; French National Centre for Scientific Research, FRANCE

## Abstract

Epidemiologic studies have demonstrated that some bacteria colonization or infections in early-life increased the risk for subsequent asthma development. However, little is known about the mechanisms by which early-life bacterial infection increases this risk. The aim of this study was to investigate the effect of neonatal *Streptococcus pneumoniae* infection on the development of adulthood asthma, and to explore the possible mechanism. A non-lethal *S*. *pneumoniae* lung infection was established by intranasal inoculation of neonatal (1-week-old) female mice with D39. Mice were sensitized and challenged with ovalbumin in adulthood to induce allergic airways disease (AAD). Twenty-four hours later, the lungs and bronchoalveolar lavage fluid (BALF) were collected to assess AAD. Neonatal *S*. *pneumoniae* infection exacerbated adulthood hallmark features of AAD, with enhanced airway hyperresponsiveness and increased neutrophil recruitment into the airways, increased Th17 cells and interleukin (IL)-17A productions. Depletion of IL-17A by i.p. injection of a neutralizing monoclonal antibody reduced neutrophil recruitment into the airways, alleviated airway inflammation and decreased airway hyperresponsiveness. Furthermore, IL-17A depletion partially restored levels of inteferon-γ, but had no effect on the release of IL-5 or IL-13. Our data suggest that neonatal *S*. *pneumoniae* infection may promote the development of adulthood asthma in association with increased IL-17A production.

## Introduction

Asthma is a common disease in children and the origin of the majority of adult cases, indicating that childhood events have an important role in asthma pathogenesis [1–3]. Childhood is an important period for the maturation of the immune system, and specific infections may alter immunologic programming, which plays a critical role in the progression of allergic airways disease (AAD) in adulthood.

Despite remarkable progress in our understanding of the pathogenesis of asthma, the initiating events have not been elucidated. Studies have demonstrated that viral infections in childhood promote subsequent development of asthma [4–7]. Recent studies suggest some bacterial infection may also have an important role in asthma pathogenesis [8,9]. Bronchial microbial florae in asthmatic patients are disturbed compared to healthy controls [10]. Some young children with acute episodes of wheezing have bacterial infections that are closely associated [11]. Neonates colonized with *Streptococcus pneumoniae*, *Haemophilus influenzae*, *Moraxella catarrhalis*, or a combination of these three, have an increased risk for recurrent wheezing and asthma [12], and these bacteria are consistently associated with exacerbations of asthma in children [13]. *S*. *pneumoniae* is the most common bacterial pathogen of community-acquired pneumonia, meningitis and sepsis, especially in young children [14,15]. Preston et al. [16] showed that *S*. *pneumoniae* infection in adult mice induces T_regs_ cells and suppresses AAD. Whether *S*. *pneumoniae* infection in neonatal mice can induce different immune responses is unclear.

Interleukin (IL)-17A is an important mediator of neutrophilic inflammation, and is elevated in the sputum of asthmatic patients with increased neutrophilia [17]. IL-17A also plays a critical role in host protection against bacterial infections, indicating its potential role in the pathogenesis of bacteria-induced neutrophilic asthma [18,19]. In this study, we investigated the effect of neonatal *S*. *pneumoniae* infection on AAD in an adult mouse model with or without IL-17A depletion.

## Materials and Methods

### Animals

Pathogen-free pregnant BALB/c dams (6–8 wk of age) were housed in individually filtered cages, maintained on a 12-h light/dark cycle with an ovalbumin (OVA)-free diet, under a constant room temperature (24°C). Adequate amounts of sterile animal food and water were provided. Cages, food, bedding, and water were sterilized before use. The Institutional Animal Care and Research Advisory Committee at Chongqing Medical University approved this study. The use of animals in these experiments was in accordance with the guidelines issued by the Chinese Council on Animal Care.

### Non-lethal neonatal *S*. *pneumoniae* infection and AAD induction

**Fig 1:**
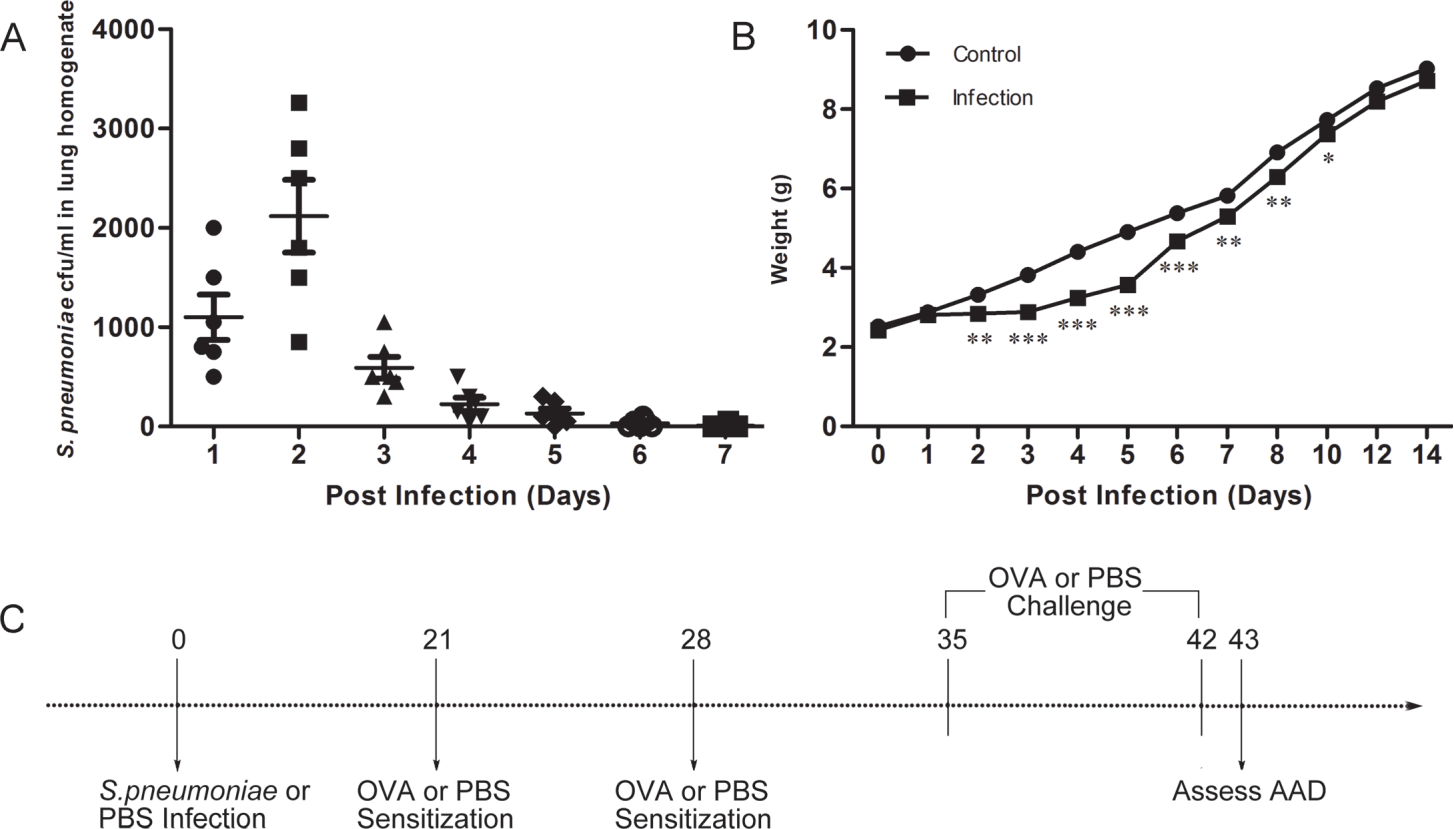
Establishment of neonatal *S*. *pneumoniae* infection model and schematic of study protocol. Neonatal BALB/c mice were divided into the following groups: infected non-allergic (Neo), infected allergic (Neo/OVA), uninfected allergic (OVA), and uninfected non-allergic (control). Mice were infected intranasally with *S*. *pneumoniae* or phosphate-buffered saline (PBS) on day 0 (1 week-old). The *S*. *pneumoniae* clearance time (A) and the body weight (B) were monitored. Mice were sensitized by an i.p. injection of ovalbumin (OVA) or PBS on days 21 and 28, and challenged with aerosolized OVA or PBS to induce allergic airways disease (AAD) on days 35–42. Key features of AAD were characterized within 24 h after the final challenge (on day 43) (C).

Non-lethal neonatal *S*. *pneumoniae* infection was established according to the procedures described in our previous study [20]. Briefly, *S*. *pneumoniae* (D39) were plated onto tryptic soy broth (Pangtong, China), cultured for 8–10 h at 37°C in a 5% CO_2_ atmosphere, washed, and suspended in sterile phosphate-buffered saline (PBS). Conscious neonatal (Neo, 1-wk-old) female BALB/c mice were infected intranasally with 2 × 10^7^ colony-forming units (CFU) of *S*. *pneumoniae* (D39) in 5 μL of PBS. The *S*. *pneumoniae* clearance time and the body weight were monitored. *S*. *pneumoniae* in lungs were cleared away within 7 days (Fig. 1A), and the body weight was recovered within 14 days (Fig. 1B). Mice were divided into the following groups: infected non-allergic (Neo), infected allergic (Neo/OVA), uninfected allergic (OVA), and uninfected non-allergic (control). To induce AAD, mice in the Neo/OVA and OVA groups were sensitized with i.p. injections of 100 μg OVA (Sigma-Aldrich, St. Louis, MO, USA) diluted in 50% aluminum hydroxide gel (Sigma-Aldrich) for a total volume of 200 μL on days 21 and 28 following inoculation. From days 35–42, mice were exposed to 1% OVA aerosols for 30 min/d. Neo mice and controls were simultaneously sensitized and challenged with sterile PBS. AAD was assessed within 24 h after the final challenge (Fig. 1C). Each experiment was repeated three times for a total of four to eight mice per group.

### Airway hyperresponsiveness (AHR)

AHR to methacholine was assessed using a mouse plethysmograph as previously described [21–23]. Briefly, 24 h after the final challenge, AHR was assessed in conscious, spontaneously breathing mice by means of whole-body plethysmography (Emca Technologies, Allmedicus, France). Each mouse was exposed to nebulized PBS followed by incremental doses of nebulized methacholine (3.125, 6.250, 12.500, 25.000, and 50.000 mg/mL; Sigma-Aldrich) for 3 min, and mean Penh values were recorded 5 min after administration of each dose; Penh = peak expiratory flow / peak inspiratory flow × pulmonary airflow resistance.

### Histopathology of the lungs

Twenty-four hours after the final OVA challenge, mice were sacrificed by a lethal dose of 10% chloral hydrate (0.3 mL/100 g, i.p.) to harvest the lungs. Formalin-fixed lungs were dissected and embedded in paraffin. Four-micron-thick sections were cut and stained with hematoxylin and eosin (Sigma-Aldrich) according to the manufacturer’s instructions. Images were captured under a Nikon Eclipse E200 microscope connected to a Nikon Coolpix 995 camera (Nikon, Tokyo, Japan). The degree of airway inflammatory cell infiltration was scored in a single-blind fashion to reduce evaluator bias. Lung lesions were scored semi-quantitatively as previously described [24].

### Bronchoalveolar lavage and cell counting

Within 24 h after the final challenge, mice were anesthetized with 10% chloral hydrate (0.1 mL/100 g, i.p.). The trachea was cannulated, and bronchoalveolar lavage fluid (BALF) was obtained by flushing the lungs twice with 1 mL PBS. Total cell numbers in the BALF were counted using microscopy. Differential cell counts were performed under Wright-Giemsa staining and based on standard morphologic and staining characteristics of at least 250 cells per sample. The supernatant was stored at −80°C. All slides were characterized by a single-blinded examiner to reduce evaluator bias.

### BALF cytokine measurements

Cytokine concentrations in BALF were measured with commercial enzyme-linked immunosorbent assay (ELISA) kits according to the manufacturer's instructions. ELISA kits were used to measure interferon (IFN)-γ (Xinbosheng, Shenzhen, China), IL-5, IL-10 and transforming growth factor (TGF)-β (Sizhengbai, Beijing, China), and IL-17A and IL-13 (eBioscience Inc., San Diego, CA, USA).

### Flow cytometric analysis of lung CD4^+^ T cells

The lungs were minced and incubated for 20 min at 37°C in 1 mL of sterile PBS containing 0.2% collagenase I (Sigma-Aldrich). Single pulmonary cell suspensions were obtained by forcing tissue through a 70 μm cell filter (Becton, Dickinson and Company, Franklin Lakes, NJ, USA). Erythrocytes were lysed, and the remaining cells were resuspended in RPMI 1640 medium containing 10% fetal bovine serum. A single-cell suspension from the lung (2 × 10^6^ cells/mL) was incubated for 4–6 h at 37°C and 5% CO_2_ in six-well flat-bottom plates (Nalgene of Thermo Fisher Scientific, Waltham, MA, USA) in 1 mL medium containing phorbol 12-myristate 13-acetate (50 ng/mL; Sigma-Aldrich), ionomycin (500 ng/mL; Sigma-Aldrich) and GolgiPlug-containing brefeldin A (Becton, Dickinson and Company). The cells were then harvested, washed, pretreated with an Fc blocker, and subsequently stained for surface-associated CD4 (anti–CD4-FITC; Pharmingen of Becton, Dickinson and Company), CD3 (anti–CD3-Cy7; Pharmingen), or CD25 (anti–CD25-PE; eBioscience Inc.). To detect the subsets of CD4^+^ T cells in lungs, the cells were stained for intracellular IFN-γ (anti–IFN-γ-PerCP-Cy5.5; Pharmingen), IL-17A (anti–IL-17A-PE; Pharmingen), IL-4 (anti–IL-4-APC; Pharmingen), and FOXP3 (anti–FOXP3-PE-Cy5; eBioscience Inc.), and detected by flow cytometry (FACS Canto; Becton, Dickinson and Company). The data were analyzed with CellQuest software (Becton, Dickinson and Company).

### IL-17A blockade during OVA-induced AAD

In some experiments, IL-17A was blocked according to the method previously described by Essilfie et al. [9]. Briefly, IL-17A was depleted on days 34 and 36 by i.p. injection of a monoclonal anti-IL-17A neutralizing antibody (clone 50104, rat IgG2a, 100 μg/mouse; R&D Systems, Inc., Minneapolis, MN, USA). Features of AAD were assessed on day 43. Control groups received an IgG2a isotype control antibody (R&D Systems, Inc.).

### Statistics

Results were analyzed using GraphPad Prism (version 5.0; GraphPad, La Jolla, CA, USA) and expressed as mean ± the standard error. Results were interpreted using either one-way analysis of variance (ANOVA) and Tukey’s post-test or a two-way ANOVA with a Bonferroni’s post-test. Differences were considered statistically significant when *P* < 0.05.

## Results

### Neonatal *S*. *pneumoniae* infection promotes OVA-induced neutrophilic inflammation in a BALB/c mouse model

**Fig 2:**
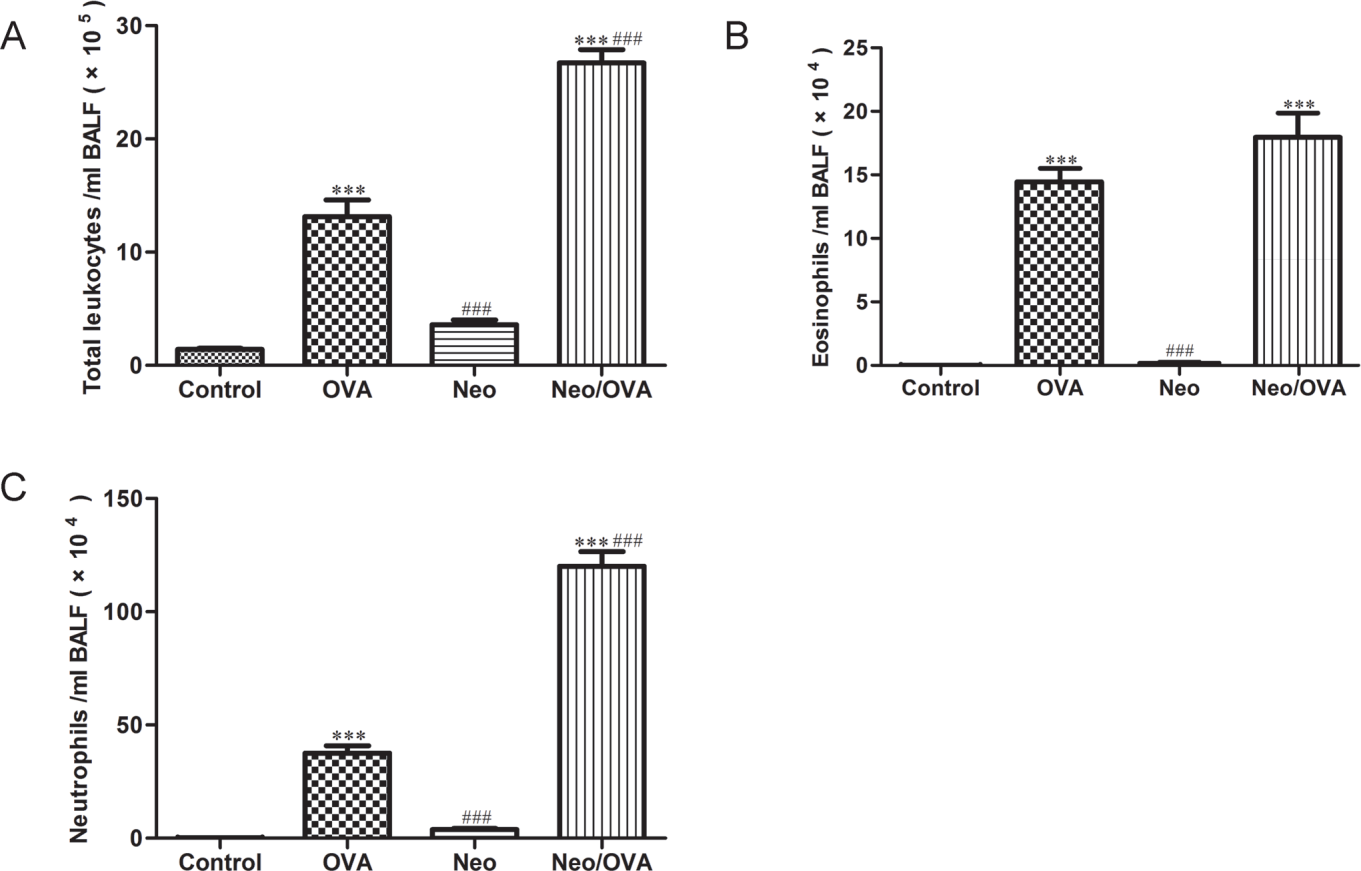
Neonatal *S*. *pneumoniae* infection enhances ovalbumin (OVA)-induced leukocyte and neutrophil infiltration of the airways. Total cells (A), eosinophils (B), and neutrophils (C) were counted from bronchoalveolar lavage fluid (BALF) collected 24 h after the final challenge. OVA, uninfected, allergic; Neo/OVA, neonatal infected, allergic; Neo, neonatal infected, non-allergic; Control, uninfected, non-allergic. Data are shown as mean ± standard error from three separate experiments (*n* = 6–8 mice/group); ***P < 0.001 *vs*. controls; ^###^ *P* < 0.001 *vs*. OVA.

To determine the effect of neonatal *S*. *pneumoniae* lung infection on adulthood airway inflammation, the number of inflammatory cells in BALF was counted. Total numbers of inflammatory cells (Fig. 2A) and eosinophils (Fig. 2B) in the OVA group were elevated > 10-fold compared to controls (13.13 ± 1.49 *vs*. 1.42 ± 0.11 and 14.41 ± 1.09 *vs*. 0.03 ± 0.016, respectively; *P*s < 0.001). Notably, the neonatal *S*. *pneumoniae* infection in the Neo/OVA mice enhanced total numbers of inflammatory cells and neutrophils (Fig. 2A,C) compared with the uninfected OVA group (26.71 ± 1.17 *vs*. 13.13 ± 1.49 and 120.10 ± 6.43 *vs*. 37.52 ± 3.34, respectively; *P*s < 0.001), while the number of eosinophils did not differ (Fig. 2B).

### Neonatal *S*. *pneumoniae* infection aggravates OVA-induced lung pathology

**Fig 3:**
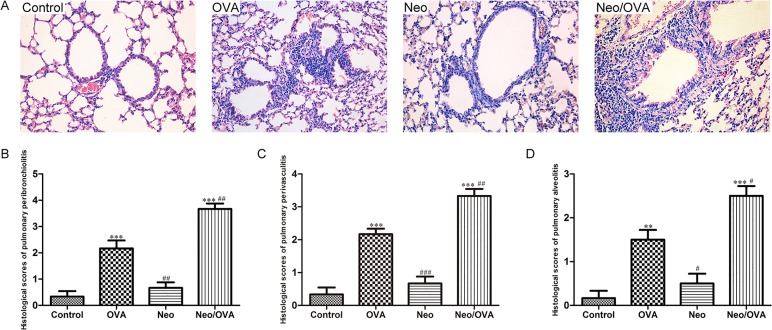
*S*. *pneumoniae* infection is associated with enhanced inflammatory tissue pathology of allergic airways disease. Hematoxylin and eosin staining of lung samples from uninfected, allergic (OVA), neonatal infected, allergic (Neo/OVA), neonatal infected, non-allergic (Neo), and uninfected, non-allergic (control) mice (A) (200×). Histologic scores of pulmonary peribronchiolitis (B), pulmonary perivasculitis (C), and pulmonary alveolitis (D). Data are reported as mean ± standard error from three separate experiments (*n* = 6–8 mice/group). **P < 0.01, ***P < 0.001 *vs*. control; ^#^ *P* < 0.05, ^##^ *P* < 0.01, ^###^ *P* < 0.001 *vs*. OVA.

We next examined the lung pathology following OVA sensitization and challenge. OVA challenge led to a dense peribronchiolar and perivascular infiltrate of inflammatory cells. In the Neo/OVA group, lung tissue inflammation was more severe with greater infiltration of inflammatory cells compared with the OVA group (Fig. 3A). The inflammation scores for pulmonary peribronchiolitis, pulmonary perivasculitis, and pulmonary alveolitis in the Neo/OVA group mice were almost double those in the OVA group (*P*s < 0.01) (Fig. 3B-D).

### Neonatal *S*. *pneumoniae* infection enhances AHR during AAD

**Fig 4:**
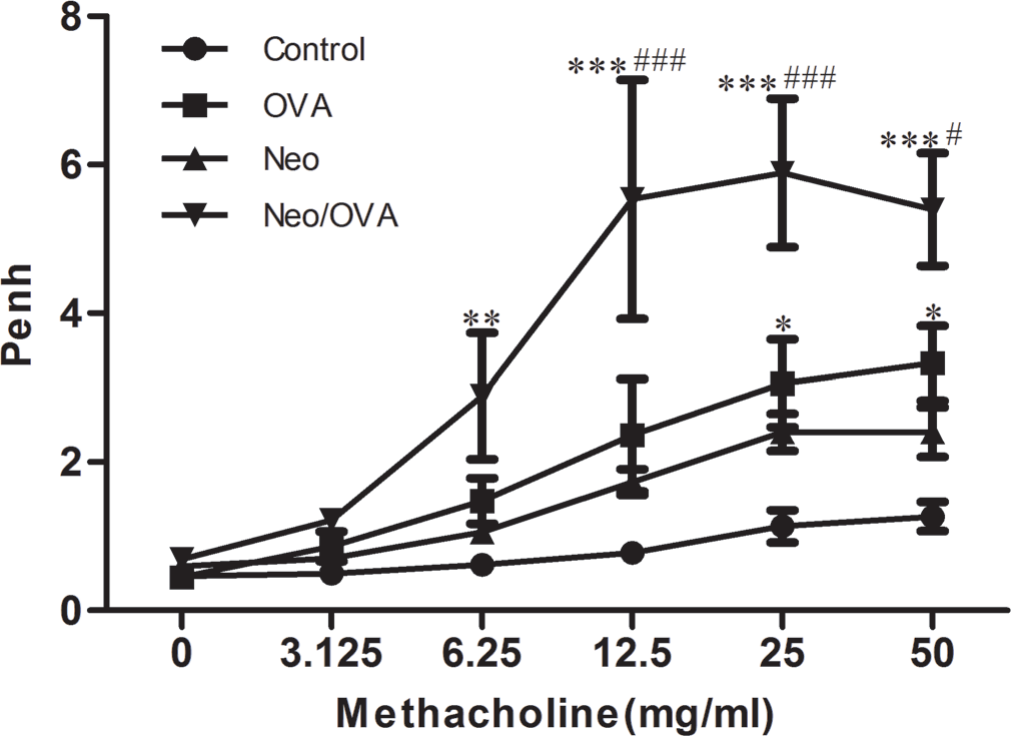
Neonatal *S*. *pneumoniae* infection enhances ovalbumin-induced airway hyperresponsiveness. Whole-body plethysmography in uninfected, allergic (OVA), neonatal infected, allergic (Neo/OVA), neonatal infected, non-allergic (Neo), and uninfected, non-allergic (control) mice was conducted 24 h following challenge with methacholine. Data are reported as mean ± standard error from three separate experiments (*n* = 6–8 mice/group); *P < 0.05, **P < 0.01, ***P < 0.001 *vs*. control; ^#^ *P* < 0.05, ^###^ *P* < 0.001 *vs*. OVA.

AHR was evaluated 24 h after the final challenge by the calculation of Penh values (i.e., enhanced respiratory pausing). OVA sensitization and challenge resulted in increased AHR (Fig. 4). The Penh values for the OVA group were higher than those for controls at methacholine concentrations between 25.0 and 50.0 mg/mL (*P*s < 0.001). Furthermore, the Neo/OVA group had a significantly higher Penh value than the OVA and control groups (*P*s < 0.001).

### Neonatal *S*. *pneumoniae* infection promotes IL-17A production during AAD

**Fig 5:**
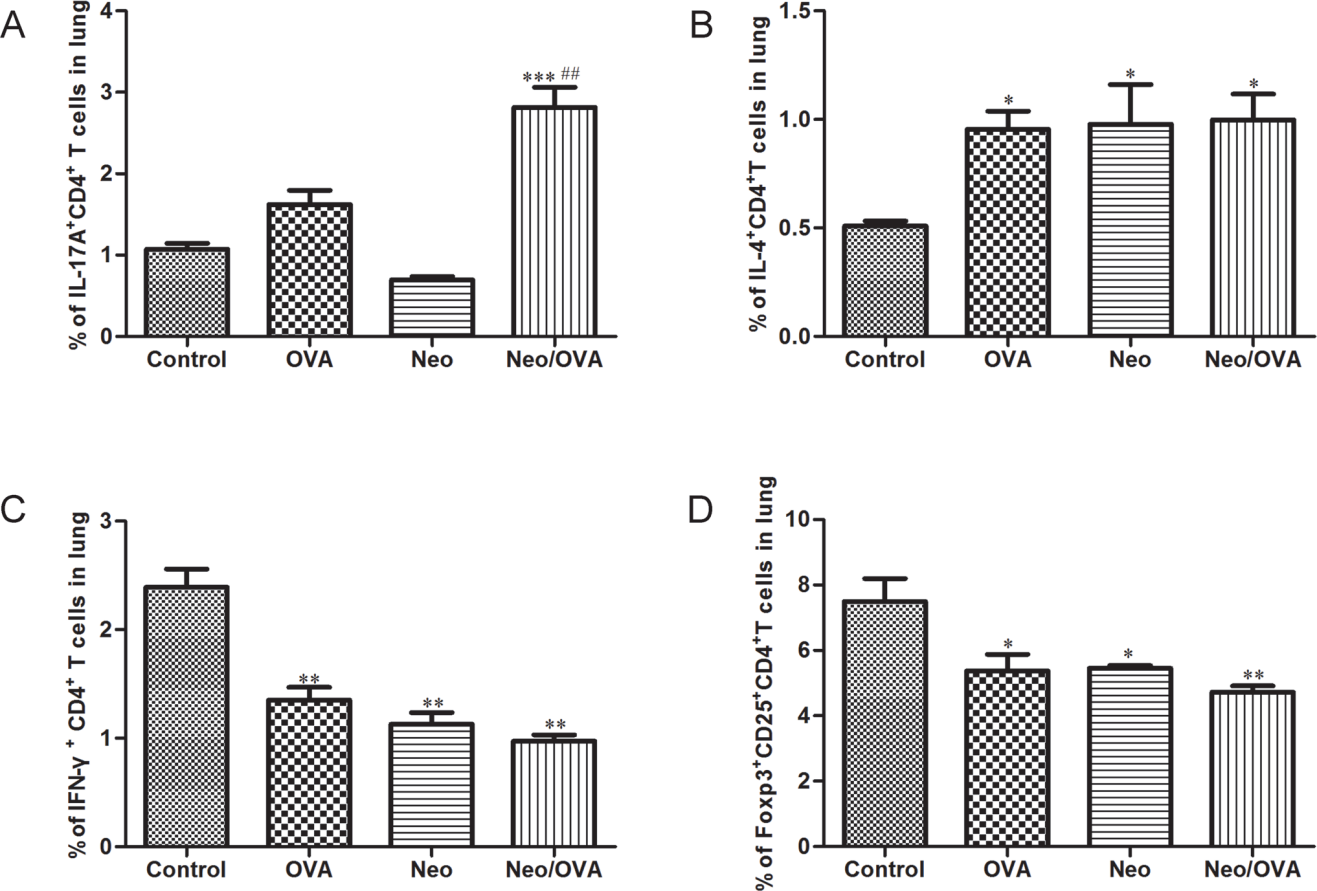
Neonatal *S*. *pneumoniae* infection-induced allergic airways disease in adulthood correlates with Th17 but not Th2 cells. Th17 (A), Th2 (B), Th1 (C), and FOXP3^+^ T_reg_ (D) subsets of CD4^+^ T cells were measured in the lungs of uninfected, allergic (OVA), neonatal infected, allergic (Neo/OVA), neonatal infected, non-allergic (Neo), and uninfected, non-allergic (control) mice by flow cytometry. Data are reported as mean ± standard error from three separate experiments (*n* = 6–8 mice/group); *P < 0.05, **P < 0.01, ***P < 0.001 *vs*. controls; ^##^ *P* < 0.01 *vs*. OVA.

We first investigated the effects of neonatal *S*. *pneumoniae* lung infection on CD4^+^ T cell production during ADD. Interestingly, the production of Th17 cells in the Neo/OVA group was significantly higher than in the OVA group (*P* < 0.001) (Fig. 5A). However, there were no differences in the production of Th2, Th1, and FOXP3^+^ T_reg_ between the Neo/OVA and the OVA groups (Fig. 5B-D).

**Fig 6:**
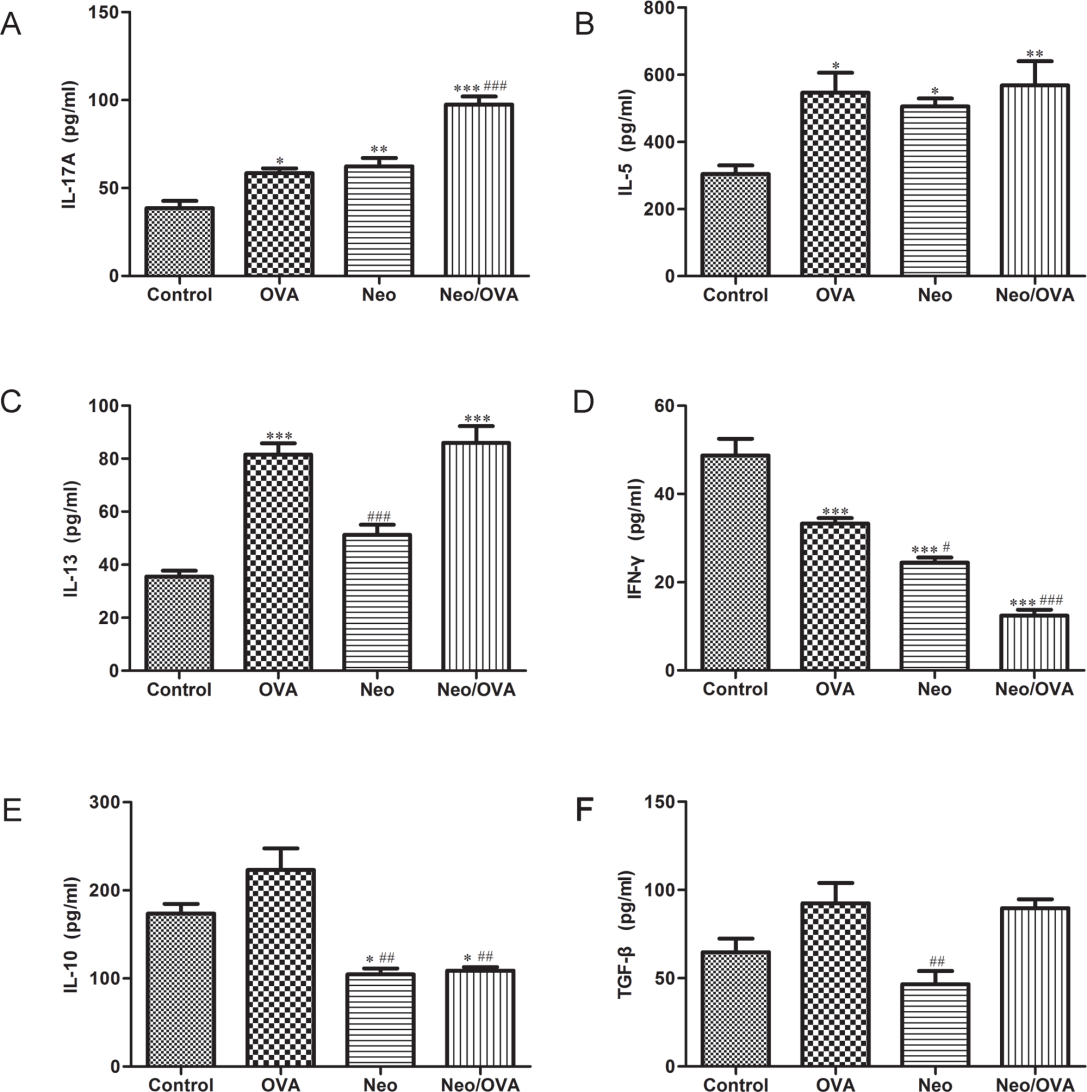
Airway inflammation in mice with neonatal *S*. *pneumoniae* infection correlates with Th17 cytokine production. Cytokine levels of interleukin (IL)-17A (A), IL-5 (B), IL-13 (C), interferon (IFN)-γ (D), IL-10 (E), and transforming growth factor (TGF)-β (F) in the bronchoalveolar lavage fluid of uninfected, allergic (OVA), neonatal infected, allergic (Neo/OVA), neonatal infected, non-allergic (Neo), and uninfected, non-allergic (control) mice were measured by ELISA. Data are reported as mean ± standard error from three separate experiments (*n* = 6–8 mice/group); *P < 0.05; **P < 0.01, ***P < 0.001 *vs*. controls; ^#^ *P* < 0.05, ^##^ *P* < 0.01, ^###^ *P* < 0.001 *vs*. OVA.

Next, we detected cytokine concentrations in BALF. As expected, IL-17A production in the Neo/OVA group was significantly higher (two-fold) than in the OVA group *(P* < 0.001) (Fig. 6A), though the levels of IL-5 and IL-13 were similar (Fig. 6B,C). The levels of IFN-γ and IL-10 (Fig. 6D,E) in the Neo/OVA group were half of those observed in the OVA group (*P*s < 0.001), while TGF-β was similar (Fig. 6F).

### Neonatal *S*. *pneumoniae* infection exacerbates OVA-induced AAD in association with an IL-17A response

**Fig 7:**
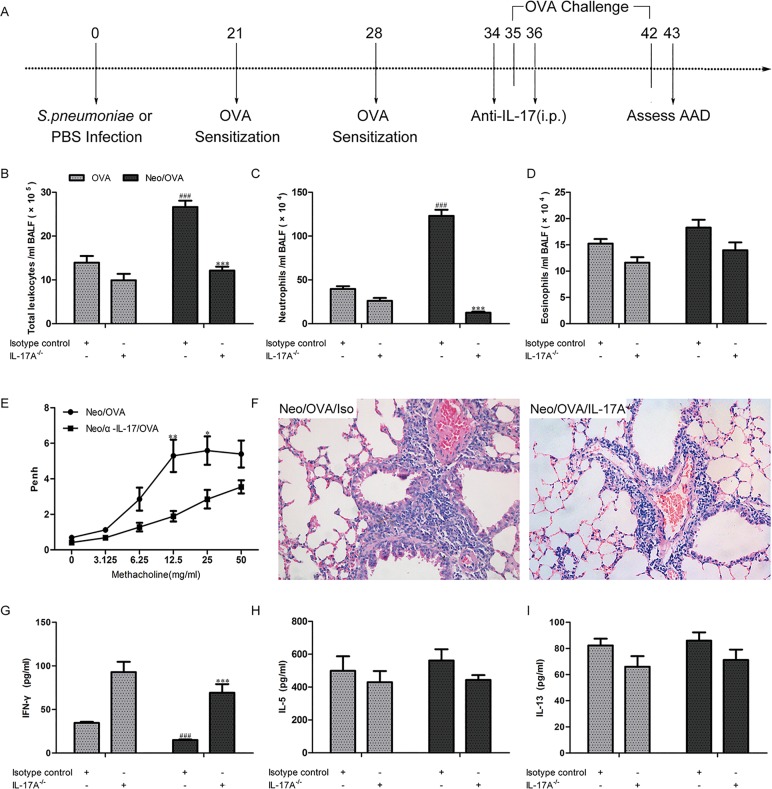
S. pneumoniae infection-induced interleukin (IL)-17A is responsible for the aggravated allergic airways disease (AAD). Anti-IL-17A monoclonal antibody was administrated by i.p. injection on days 34 and 36, and features of AAD were assessed on day 43 (A). The influx of total inflammatory cells (B), neutrophils (C), eosinophils (D), airway hyperresponsiveness (E), tissue pathology (F), and the levels of interferon (IFN)-γ (G), IL-5 (H), IL-13 (I) in bronchoalveolar lavage fluid (BALF) were assessed. Data are reported as mean ± standard error from three separate experiments (*n* = 4–6 mice/group); ***P < 0.001 *vs*. isotype control; ^*###*^ *P* < 0.001 *vs*. OVA. OVA = uninfected, allergic, Neo/OVA = neonatal infected, allergic.

To determine whether IL-17A mediates neonatal *S*. *pneumoniae*-induced airway inflammation, IL-17A was depleted in the Neo/OVA and OVA mice during AAD (Fig. 7A). As expected, IL-17A depletion in the Neo/OVA mice with an IL-17A-blocking monoclonal antibody significantly reduced total inflammatory cell and neutrophil recruitment into the BALF as compared to isotype antibody treatment (*P*s < 0.001) (Fig. 7B,C). Consistent with our results that eosinophils were unaffected in neonatal *S*. *pneumoniae* infection, eosinophil numbers were similar in mice treated with either anti-IL-17A or isotype antibody (Fig. 7D). Anti-IL-17A treatment decreased AHR (Fig. 7E) and alleviated airway inflammation (Fig. 7F). Anti-IL-17A treatment also partially restored IFN-γ (Fig. 7G) production, but had no effect on IL-5 (Fig. 7H) or IL-13 production (Fig. 7I).

## Discussion

In this study, we investigated the effect of non-lethal neonatal *S*. *pneumoniae* lung infection on the development of allergic asthma in adulthood. We demonstrate that neonatal *S*. *pneumoniae* lung infection promotes the recruitment of OVA-induced neutrophils into the airways, aggravates airway inflammation and increases AHR in adulthood. Furthermore, IL-17A depletion alleviates airway inflammation, decreases AHR, and reduces airway neutrophil recruitment. Thus, neonatal *S*. *pneumoniae* lung infection exacerbates the hallmark features of AAD in adulthood, which may be associated with increased IL-17A production.

*S*. *pneumoniae* has been used to investigate *S*. *pneumoniae*-induced infection and host-bacteria-allergen interactions in susceptible BALB/c mice [25]. Studies have found that adulthood *S*. *pneumoniae* infection, or treatment with components of or killed organisms, can suppress the hallmark features of AAD by inducing T_regs_ cells in mice [16,26]. Consistent with these results, we found that adulthood *S*. *pneumoniae* infection can suppress Th2 cells and AAD by inducing T_regs_ cells (data not shown), while neonatal *S*. *pneumoniae* infection promotes subsequent adulthood allergic asthma development, characterized by neutrophil recruitment into the airways and increased Th17 and IL-17A production. Larsen et al. [27] showed an abnormal immune response to airway-colonizing pathogenic bacteria in early-life may lead to chronic airway inflammation and childhood asthma. Horvat et al. [28] stated early-life, but not adult, chlamydial infection promotes subsequent development of allergic asthma. These data indicate that the immune response to some pathogens in childhood may be different from that in adulthood. Thus, the age of infection may have a crucial role in determining the nature of the effects, particularly in the development of subsequent allergic asthma.

In this study, Th17 cells in the lung, IL-17A and neutrophils in BALF were significantly higher in infected compared to uninfected allergic mice, while Th2 cells and their associated cytokines (IL-5, IL-13) and the number of eosinophils were similar. IL-17A, mainly produced by Th17 cells, is implicated in the pathogenesis of several inflammatory conditions [29]. Lu etal. [30] stated that IL-17A plays critical role in host protection against *S*. *pneumoniae* colonization and infection. Additional studies indicate that enhanced IL-17A levels correlate with increased AHR in asthmatics and allergic asthma in mice [31,17]. IL-17A can induce structural lung cells to secrete proinflammatory cytokines and neutrophil chemotactic proteins, thereby inducing neutrophil infiltration [32]. Thus, IL-17A has a pivotal role in the pathogenesis of asthma. Consistent with this notion, depletion of IL-17A reduced the aggravation features of AAD by neonatal *S*. *pneumoniae* infection. IL-17A depletion significantly inhibited neutrophil recruitment into the airways, decreased airway inflammation and AHR, with no significant effect on eosinophil recruitment or IL-5 and IL-13 production, suggesting that IL-17A-mediated neutrophilic inflammation plays an important role in the promotion of asthma development in adulthood by neonatal *S*. *pneumoniae* lung infection. These findings are consistent with those of Essilfie et al. [9], who demonstrated that *H*. *influenzae* infection drives IL-17-mediated development of neutrophilic AAD.

Neonatal *S*. *pneumoniae* infection also inhibited IFN-γ production, which was partially restored by IL-17A blockade. Newcomb et al. [33] showed that IFN-γ is a negative regulator of IL-17A expression in a model of respiratory syncytial virus infection during allergic airway inflammation. Whether IFN-γ is involved in regulating IL-17A production in neonatal *S*. *pneumoniae* infection-induced AAD in adults requires further investigation.

## Conclusions

Neonatal *S*. *pneumoniae* lung infection may promote the development of allergic asthma in adulthood in association with enhanced IL-17A production.
